# The Minimal *Bacillus subtilis* Nonhomologous End Joining Repair Machinery

**DOI:** 10.1371/journal.pone.0064232

**Published:** 2013-05-17

**Authors:** Miguel de Vega

**Affiliations:** Instituto de Biología Molecular “Eladio Viñuela” (CSIC), Centro de Biología Molecular “Severo Ochoa” (CSIC-UAM), Cantoblanco, Madrid, Spain; Saint Louis University, United States of America

## Abstract

It is widely accepted that repair of double-strand breaks in bacteria that either sporulate or that undergo extended periods of stationary phase relies not only on homologous recombination but also on a minimal nonhomologous end joining (NHEJ) system consisting of a dedicated multifunctional ATP-dependent DNA Ligase D (LigD) and the DNA-end-binding protein Ku. *Bacillus subtilis* is one of the bacterial members with a NHEJ system that contributes to genome stability during the stationary phase and germination of spores, having been characterized exclusively *in vivo*. Here, the *in vitro* analysis of the functional properties of the purified *B. subtilis* LigD (*Bsu*LigD) and Ku (*Bsu*Ku) proteins is presented. The results show that the essential biochemical signatures exhibited by *Bsu*LigD agree with its proposed function in NHEJ: i) inherent polymerization activity showing preferential insertion of NMPs, ii) specific recognition of the phosphate group at the downstream 5′ end, iii) intrinsic ligase activity, iv) ability to promote realignments of the template and primer strands during elongation of mispaired 3′ ends, and v) it is recruited to DNA by *Bsu*Ku that stimulates the inherent polymerization and ligase activities of the enzyme allowing it to deal with and to hold different and unstable DNA realignments.

## Introduction

Among the diverse types of DNA damage, DNA double-strand breaks (DSBs) are the most hazardous, being lethal to dividing cells if they are not repaired in a timely fashion [1]–[4]. Two main pathways deal with these lesions: the error-free homologous recombination (HR), in which a second intact double-stranded copy provides the template for DNA synthesis across the break; and the nonhomologous end joining (NHEJ), active mainly when one chromosomal copy is available, and whereby the DNA ends are directly rejoined. This pathway can be either faithful, if the DNA ends are ligated directly, or error-prone, if the ends are processed by nucleases or polymerases before being sealed by a dedicated DNA ligase [reviewed in [5]].

The presence of the NHEJ was first described in eukaryotes being the main DSB repair pathway operating during the G1 phase of the cell cycle [6], [7]. Briefly, DNA ends at DSBs are threaded through the open-ring structure of the Ku70/80 heterodimer (Yku70/Yku80 in yeast), allowing their alignment and protection against nucleases [8]–[10]. Once bound to DNA, in higher eukaryotes Ku recruits the DNA-dependent protein kinase catalytic subunit [4], [11] and both carry out the synapsis of the broken ends. In yeast, the recruited Mre11/Rad50/Xrs2 complex (MRX), required for NHEJ, has been also proposed to bind and connect two DNA molecules [1]. Those termini are further processed by nucleases (Artemis, Fen1), phosphatases and kinases generating gapped intermediates filled by a DNA polymerase belonging to family X (λ, µ and yeast Pol IV) [2], [3], [12]–[14]. Finally, the complex Ligase IV/XRCC4/XLF (Dnl4/Lif1/Nej1 in yeast) joins the ends together [1], [15].

The identification in bacterial genomes of genes homologous to the eukaryotic Ku led to the discovery of a NHEJ pathway in these organisms [16]–[22]. Bacterial Ku homologues, typically present as single genes whose product would function as a homodimer, are usually genetically associated to putative ATP-dependent DNA ligase genes (Ligase D, LigD) [16], [17], [23], [24]. In many bacteria, as in *Mycobacterium tuberculosis*, LigD contains a phosphoesterase, polymerase and ligase domain that could account for the end processing, gap filling and sealing steps during NHEJ [18], [20], [25] (see scheme in Figure S1). Extensive biochemical characterization of LigD and Ku proteins, mainly those from *M. tuberculosis* and *M. smegmatis*, as well as the impact that their absence has in the repair of DSBs, has led to propose recently how the minimal two-component NHEJ operates in bacteria [19], [21], [22], [26]. Thus, at first Ku binds to both sides of the DSB and recruits LigD whose polymerization domain recognizes specifically the 5′P termini, mediating the synapsis event required for end-joining. If after microhomology pairing non-extendable 3′ termini were present, they would be resected by the phosphodiesterase/phosphomonoesterase activity of LigD and further elongated by its polymerization activity. The nicks that result after filling those gaps would be finally sealed by the ligase activity, completing the break repair [26]. Although the bacterial NHEJ was originally envisaged to rely only on Ku and LigD factors, novel bacterial Ku partners, potentially involved in this repair pathway, have been identified, as mycobacterial UvrD1, an ATPase with a Ku-dependent unwinding activity of 3′-tailed DNA duplexes [27]. Additionally, mycobacterial Sir-2 protein has been recently shown to interact directly with both, Ku and LigD [28]. This fact, together with the marked sensitivity to ionizing radiation exhibited by *Sir-2* deficient cells, provided strong evidence supporting the involvement of this protein in mycobacterial NHEJ together with Ku and LigD. Finally, genetic evidences implicated also mycobacterial LigC in a minor pathway of Ku-dependent NHEJ [21].

As other soil organisms, the gram positive bacterium *Bacillus subtilis* can survive to adverse growth conditions, as nutrient deprivation, low temperature, acidity, dryness or high osmolarity by forming an haploid endospore that will germinate and start a new round of vegetative growth once a favorable environment is reestablished [29], [30]. The absence of an intact copy of the bacterial chromosome to act as template for the re-synthesis of DSBs precludes the HR pathway to operate during spore germination [25], [31]. As aforementioned, the initial evidence of a NHEJ pathway in *B. subtilis* was the identification of the gene *ykoV* [32], predicted to encode for a 35-kDa protein with a significant homology to the eukaryotic Ku proteins (*Bsu*Ku) [16], [19], [24]. This gene exists in an operon including the downstream gene *ykoU* potentially encoding for a putative 70-kDa bimodular LigD (*Bsu*LigD) with a C-terminal DNA ligase catalytic domain linked to an N-terminal polymerase domain that shares similarity to the catalytic subunit of eukaryal DNA primases [16], [23], [24] (see scheme in Figure S1). These genes form part of a regulon under the control of both, the RNA-polymerase sigma factor σ^G^ and the DNA-binding protein SpoVT, and whose expression is turned on in the forespore [33]. The experimental evidences of the participation of *Bsu*LigD and *Bsu*Ku in DSB repair came from the analyses of *B. subtilis ykoV* and *ykoU* null mutants. Thus, deletion of those genes sensitized *B. subtilis* cells to ionizing radiation in the stationary phase [19] and their spores to several DNA-damaging treatments known to cause DSBs, as UV-ray, X-ray, ultrahigh vacuum and wet heat [33], [34].

Here, an initial and qualitative *in vitro* characterization of the biochemical properties of *Bsu*LigD and *Bsu*Ku proteins is presented. The results show that *Bsu*LigD has intrinsic polymerization and ligase activities that act in concert to fill and finally seal short gaps. In addition, *Bsu*Ku recruits *Bsu*LigD stimulating its inherent polymerization and ligase activities allowing the enzyme to deal with and to hold different and unstable DNA realignments.

## Materials and Methods

### Nucleotides and proteins

Unlabeled nucleotides, [γ-^32^P]ATP (3000 Ci mmol^−1^), [α-^32^P]dATP (3000 Ci mmol^−1^) and [α-^32^P]ATP (3000 Ci mmol^−1^) were obtained from Amersham Pharmacia. Restriction endonucleases and T4 polynucleotide kinase were purchased from New England Biolabs. The independent open reading frames containing the genes *ykoU* and *ykoV* from *B. subtilis* [32] were PCR amplified and further digested with NdeI and BamHI before cloning in the NdeI-BamHI digested pET-28a(+) bacterial expression vector (Novagen), which carries an N-terminal His-Tag configuration to express recombinant proteins as fusions with an N-terminal hexahistidyl for purification on Ni^+2^-affinity resins. *E. coli* BL21(DE3) cells were transformed with the resulting recombinant expression plasmids, named pET28-*Bsu*LigD and pET28-*Bsu*Ku, and such constructions were further confirmed by DNA sequencing. Expression of the His-tagged *Bsu*Ku protein was carried out in the *E. coli* strain BL21(DE3), which contains the T7 RNA polymerase gene under the control of the isopropyl β-D-thiogalactopyranoside (IPTG)-inducible lacUV5 promoter [35], [36]. Cells, previously transformed with plasmid pET28-*Bsu*Ku, were grown overnight in LB medium at 37 °C in the presence of kanamycin. Cells were diluted into the same media and incubated at 30 °C until the DO_600_ reached 0.6. Then, IPTG (Sigma) was added to a final concentration of 0.5 mM and incubation was continued for 1 h at 30°C. To express *Bsu*LigD protein, chemically competent *E. coli* SoluBL21 cells (Genlantis) were transformed with the recombinant plasmid pET28-*Bsu*LigD and grown overnight in M9 minimal media at room temperature in the presence of kanamycin. Cells were diluted into the same media until OD_600_ = 0.2 and grown at 37°C until OD_600_ = 0.4. After induction of protein expression with 1 mM IPTG, cells were incubated overnight at 20°C. In both cases, cells were collected by centrifugation for 10 min at 6,143× *g*.

#### Purification of BsuLigD

Cells were thawed and ground with alumina at 4°C. The slurry was resuspended in Buffer A (50 mM Tris-HCl, pH 7.5, 0.3 M NaCl, 7 mM β-mercaptoethanol, 5% glycerol) and centrifuged for 5 min at 650× *g*, at 4 °C to remove alumina and intact cells. The recombinant *Bsu*LigD protein was soluble under these conditions, since it remained in the supernatant after a new centrifugation for 20 min at 23430× *g*, to separate insoluble proteins from the soluble extract. The soluble extracts were loaded onto a Ni-NTA column (QIAgen) pre-equilibrated with Buffer A (0.3 M NaCl). The bound proteins were eluted with 80 mM imidazole in Buffer A (0.3 M NaCl). The eluate was further loaded onto a phosphocellulose column equilibrated with buffer A (0.12 M NaCl) and *Bsu*LigD was eluted with Buffer A (0.2 M NaCl).

#### Purification of BsuKu

Cells were thawed and ground with alumina at 4°C. The slurry was resuspended in Buffer B (50 mM Tris-HCl, pH 7.5, 0.3 M NaCl, 7 mM β-mercaptoethanol, 1 mM EDTA, 5% glycerol) and centrifuged for 5 min at 650× *g*, at 4 °C to remove alumina and intact cells. The recombinant *Bsu*Ku protein was soluble under these conditions, since it remained in the supernatant after a new centrifugation for 20 min at 23430× *g*, to separate insoluble proteins from the soluble extract. The DNA present was removed by stirring for 10 min the soluble extract containing 0.3 % polyethyleneimine (PEI) followed by centrifugation for 10 min at 23430× *g*. The resulting supernatant was precipitated with ammonium sulphate to 65 % saturation to obtain a PEI-free protein pellet. After centrifugation for 25 min at 23430× *g*, the pellet was resuspended in Buffer B without NaCl to get a final 0.1 M ammonium sulphate concentration. This fraction was loaded onto a phosphocellulose column equilibrated with buffer B (0.1 M NaCl) and the *Bsu*Ku protein was eluted with Buffer A (0.5 M NaCl). The eluate was loaded onto a Ni-NTA column (QIAgen) pre-equilibrated with Buffer A (0.5 M NaCl). The bound protein was eluted by 200 mM imidazole in Buffer A (0.3 M NaCl). Finally, both proteins, *Bsu*LigD and *Bsu*Ku were dialyzed against a buffer containing 0.2 M NaCl and 50% glycerol and stored at −70°C. Final purity of the proteins was estimated to be >90 % by SDS–PAGE followed by Coomassie blue staining. The purified proteins were further loaded onto a 4 ml glycerol gradient (15–30%) containing 50 mM Tris-HCl, pH 7.5, 20 mM ammonium sulphate, 180 mM NaCl, 1 mM EDTA, and 7 mM β-mercaptoethanol, and centrifuged at 4 °C for 24 h at 348134× *g* in a Beckman TST 60.4 Swinging rotor. After centrifugation, 23 (*Bsu*LigD) and 24 (*Bsu*Ku) fractions were collected from the bottom of the tube for further analysis.

### Oligonucleotides, DNA templates and substrates

All the oligonucleotides described bellow were obtained from Invitrogen. sp1 (5′-GATCACAGTGAGTAC), sp1p (5′-GATCACAGTGAGTAG), sp1CGG (5′- GATCACAGTGAGTACCGG), Dws(5′-OH) (5′-ACTGGCCGTCGTT), Dws(5′-P) (5′-pACTGGCCGTCGTT), sp1c+18 (5′-ACTGGCCGTCGTTCTATTGTACTCACTGTGATC), sp1c+17 (5′-ACTGGCCGTCGTTTATTGTACTCACTGTGATC), sp1c+16 (5′-ACTGGCCGTCGTTATTGTACTCACTGTGATC), sp1c+15 (5′-ACTGGCCGTCGTTTTGTACTCACTGTGATC), sp1c+14 (5′-ACTGGCCGTCGTTTGTACTCACTGTGATC), sp1c+13 (5′-ACTGGCCGTCGTT GTACTCACTGTGATC), sp1c(A)+18 (5′-ACTGGCCGTCGTTCTAT**A** GTACTCACTGTGATC), sp1c+18(G) (5′-ACTGGCCGTCGTT **G**TATTGTACTCACTGTGATC). sp1 and Dws oligos are complementary to the 3′ and 5′ underlined sequences, respectively. Oligonucleotides sp1, sp1p and sp1CGG were 5′-labeled with [γ-^32^P]ATP and T4 polynucleotide kinase. sp1 was hybridized to sp1c+18 to obtain a template/primer structure. sp1p was hybridized either to sp1c+18 or sp1c(A)+18 to obtain a template/primer structure harboring a mismatched 3′ terminus. Hybridization of sp1 and either Dws(5′-OH) or Dws (5′-P) to sp1c+18, +17, +16, +15 and +14 generated DNA molecules containing gaps of 5, 4, 3, 2 and 1 nucleotides in length, respectively. The nicked molecule was obtained by hybridizing sp1 and Dws(5′-P) to sp1c+13. The three nucleotides gapped molecules containing a mispaired 3′ end were obtained by hybridizing sp1c+16 to Dws(5′-P) and either sp1p (1 nucleotide mismatched 3′ terminus) or sp1CGG (3 nucleotides mismatched 3′ terminus). sp1CGG and Dws(5′-P) were hybridized to either sp1c+18 or sp1c+18(G) to get the 5 nucleotides gapped DNA harboring a three nucleotides mismatched 3′ end. Hybridizations were performed in the presence of 0.2 M NaCl and 60 mM Tris-HCl, pH 7.5.

### DNA polymerization on activated DNA

The incubation mixture contained, in 25 µl, 50 mM Tris-HCl, pH 7.5, 1 mM dithiothreitol (DTT), 4 % glycerol, 0.1 mg ml^−1^ of bovine serum albumin (BSA), 100 nM [α-^32^P]dATP and 100 nM of the other three dNTPs, 1.5 µg of activated calf thymus DNA (DNA treated briefly with DNase Ifor preparing substrate used in the study of DNA polymerization reactions) as a substrate and 400 ng of *Bsu*LigD in the presence of the indicated concentration of MnCl_2_ or MgCl_2_. After incubation for 20 min at 30°C, the reaction was stopped by adding 10 mM EDTA, 0.1 % SDS, and the samples were filtered through Sephadex G-50 spin columns in the presence of 0.1 % SDS. Nucleotide incorporation (nmol) was calculated by counting the Cerenkov radiation of the excluded volume.

### DNA polymerization assays on defined DNA molecules

DNA-dependent polymerization was assayed on different primer-template structures, obtained by hybridization of the indicated 5′-labeled primer (sp1, sp1p and sp1CGG) to the specified template oligonucleotide. To obtain “gapped” structures of defined length, a third oligonucleotide was also hybridized to the corresponding template oligonucleotide, as described above. The incubation mixture contained, in 12.5 µl, 50 mM Tris-HCl, pH 7.5, the indicated catalytic metal ion, 1 mM DTT, 4% (v/v) glycerol, 0.1 mg ml^−1^ BSA, 1.5 nM of the DNA molecule indicated in each case, the indicated concentrations of *Bsu*LigD and *Bsu*Ku and the specified nucleotides. After incubation for the different times at 30 °C, the reactions were stopped by adding EDTA up to 10 mM. Samples were analyzed by 8 M urea-20% PAGE and autoradiography.

#### Processivity assays

The processivity of *Bsu*LigD was analyzed at different enzyme/DNA ratios. The incubation mixture contained, in 12.5 µl, 50 mM Tris-HCl, pH 7.5, 5 mM MnCl_2_, 1 mM DTT, 4% (v/v) glycerol, 0.1 mg ml^−1^ BSA, 1.5 nM of the DNA substrate indicated, 10 µM of the specified type of nucleotide, and the indicated decreasing amounts of enzyme. When stated, 40 ng of *Bsu*Ku was also added to the reaction. After incubation for 20 minutes at 30°C, the reactions were stopped by adding EDTA up to a final concentration of 10 mM. Samples were analyzed by 8 M urea-20% PAGE and autoradiography. Processivity of polymerization was assessed by analysis of the length of replication products under decreasing DNA polymerase/DNA ratios.

### Enzyme-AMP complex formation

Reactions were performed as described [37]. The incubation mixture contained, in a final volume of 20 µl, 12 mM Tris-HCl (pH 7.5), 1 mM EDTA, 20 mM ammonium sulphate, 0.1 mg ml^−1^ BSA, 5 µM [α-^32^P]ATP (2.5 µCi), 5 mM MnCl_2_ and the indicated amount of *Bsu*LigD. After incubation for 10 min at 30°C the reactions were stopped by adding 10 mM EDTA and 0.1% SDS. The samples were then filtered through Sephadex G-50 spin columns to remove the non-incoporated ATP, and further analyzed by 12% SDS-PAGE. Label transfer to the 70-kDa *Bsu*LigD polypeptide was visualized by autoradiography of the dried gel.

## Results and Discussion

### *Bsu*LigD is endowed with a polymerization activity

**Figure 1:**
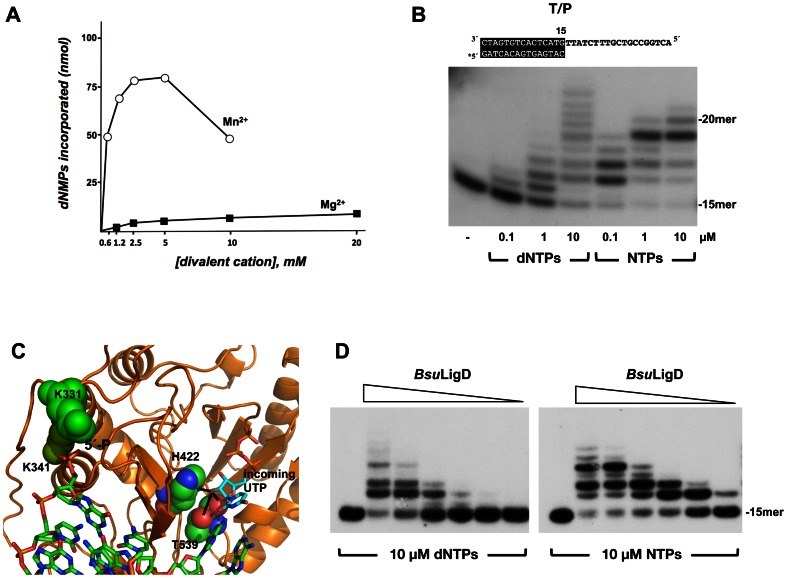
*Bsu*LigD is endowed with a polymerization activity. (A) Effect of Mg^2+^ and Mn^2+^ concentration on the polymerization activity of *Bsu*LigD. The assay was performed as described in Materials and Methods, using as substrate 1.5 µg of activated calf thymus DNA, 400 ng of *Bsu*LigD, 100 nM dNTPs and the indicated concentrations of MgCl_2_ or MnCl_2_. Polymerization activity was calculated as the amount of incorporated dNMP (nmol). (B) Polymerization activity of *Bsu*LigD on a template/primer (T/P) DNA substrate. The molecule used as substrate in the analysis is depicted on top of the figure (see also Materials and Methods). Asterisk indicates the 5′^32^P-labeled end of the primer strand. Reactions were performed as described under Materials and Methods, using 1.5 nM of each substrate, 100 ng of *Bsu*LigD, 5 mM MnCl_2_ and the indicated concentrations of nucleotides. After incubation for 20 min at 30°C, samples were analyzed by 8 M urea and 20% PAGE and autoradiography. (C) Ribbon representation of the structural model of *Bsu*LigD polymerization domain (residues 320–611). Model was provided by the homology-modelling server Swiss-Model [55]–[57], using as template the crystallographic structure of the *Mtu*LigD preternary complex [PDB code 3PKY [42]]. *Bsu*LigD residues K331 and K341, potentially involved in the specific recognition of the downstream 5′-P group are shown as lime and green space-filling spheres, respectively. The 2′-OH group of the incoming UTP is proposed to make hydrogen bonds (black lines) with *Bsu*LigD residues H422 and T539, represented as space-filling spheres. Figure was made using PyMOL software (http://www.pymol.org). (D) BsuLigD shows a distributive polymerization pattern. The assay was carried out as described in Materials and Methods, using T/P substrate depicted on top of the part B, in the presence of 10 µM of the indicated type of nucleotides and decreasing amounts of *Bsu*LigD (50, 25, 6, 2, 0.4 and 0.1 ng). After incubation for 20 min at 30°C, samples were analyzed by 8 M urea-20% PAGE and autoradiography.

The purified *Bsu*LigD was first assayed for a potential DNA polymerase activity on activated calf-thymus DNA, in the presence of increasing concentrations of either Mg^2+^ or Mn^2+^ ions. As observed in Figure 1A, although *Bsu*LigD catalyzed DNA synthesis under both conditions, it showed a preferential use of Mn^2+^ over Mg^2+^, as described for other LigDs [25], [31], [38], [39].

As shown in Figure 1B, *Bsu*LigD catalyzed the template directed addition of both dNTPs and NTPs on a template/primer structure. Although the enzyme displayed a higher efficiency in the use of NTPs respect to dNTPs, a hallmark of bacterial LigDs [18], [20], [21], [25], [31], [38]–[40], NMP incorporation was mainly limited to 1–5 nucleotides. Only a very low fraction of the primers were extended beyond the n+5 template position, strongly suggesting that *Bsu*LigD prefers a DNA to an RNA primer-terminus, as described for *Pseudomonas aeruginosa* LigD (*Pae*LigD) whose polymerization activity decreased progressively after each serial NMP incorporation [38], [41]. The explanation for the preferential utilization of NTPs came from the crystallographic structures of the polymerization domain of both, *Pae*LigD and *Mtu*LigD bound to Mn^2+^ and incoming NTP [25], [31], [42], [43]. In those complexes the 2′-OH group of the ribose sugar was hydrogen bonded to the side chain hydroxyl and backbone carboxyl oxygen of *Mtu*LigD residue T236 (*Pae*LigD residue S768) and to the δN of *Mtu*LigD residue H111 (*Pae*LigD residue H651). Those residues, conserved among the polymerization domain of bacterial LigDs [26], that would correspond to *Bsu*LigD residues T539 and H422 (see Figure 1C), could account for the preferential NMP additions exhibited by LigDs [43]. This fact has been related with the necessity to repair DSBs when the intracellular pool of dNTPs is depleted, as it occurs during the stationary phase or cellular quiescent states [18], [25]. To determine the polymerization processivity limit of *Bsu*LigD, chain length distribution was analyzed as a function of enzyme/DNA ratio. As shown in Figure 1D, the length of the products synthesized by *Bsu*LigD decreased with the enzyme/DNA ratio, in agreement with a distributive polymerization behavior. From these data it can be inferred that the enzyme dissociates from the DNA after each nucleotide incorporation step.

### *Bsu*LigD is well suited to act on short gapped DNA substrates. Specific recognition of the phosphate group at the downstream 5′ end

**Figure 2:**
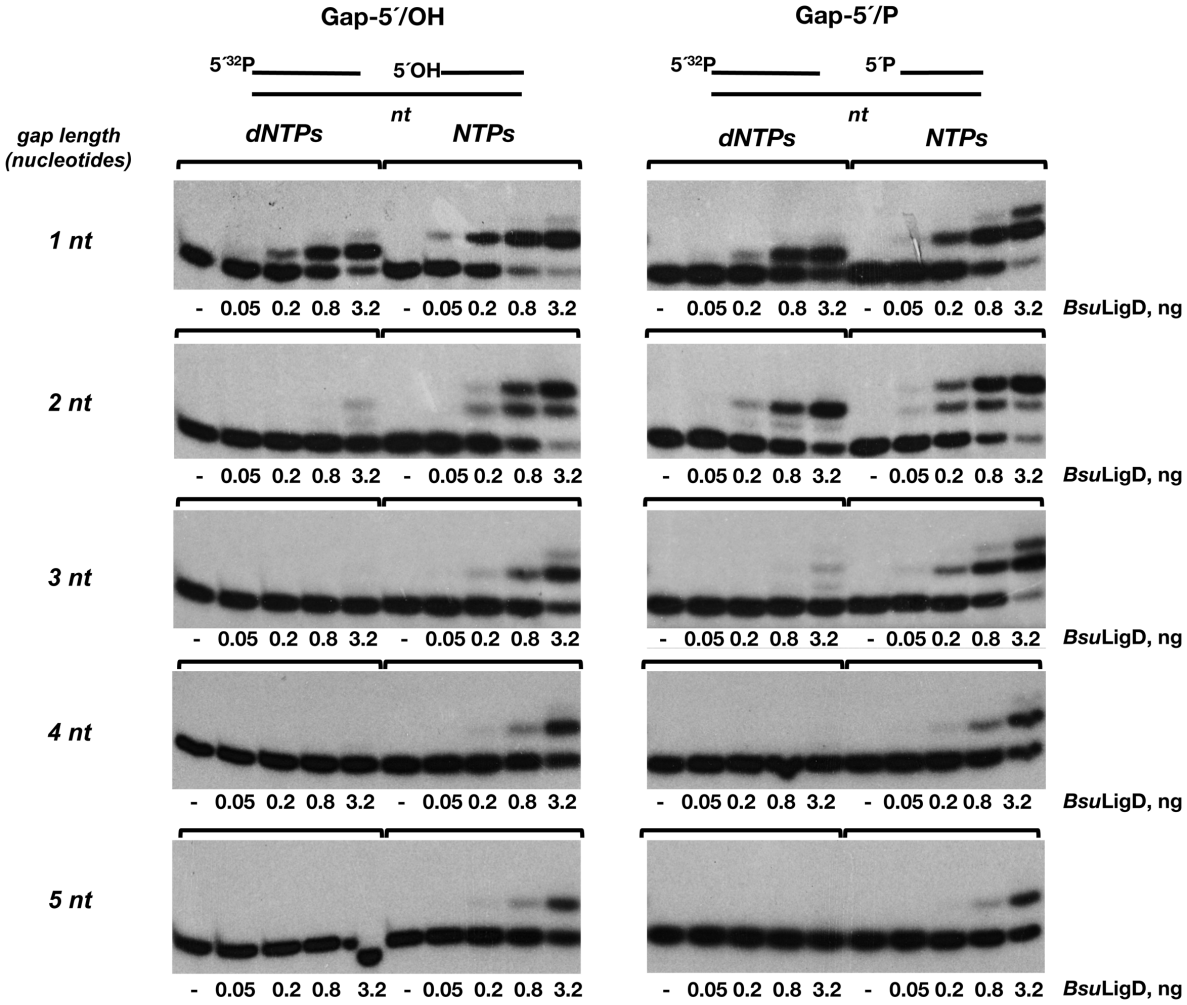
Influence of the gap length and the 5′ group of the downstream strand in the gap-filling efficiency of ***Bsu*** **LigD.** The assay was performed essentially as described in Materials and Methods. The indicated amounts of *Bsu*LigD were incubated with 2.5 nM of the indicated gapped molecule in the presence of 100 µM of the specified nucleotide and 5 mM MnCl_2_. After incubation for 5 min at 30°C, samples were analyzed by 8 M urea-20% PAGE and autoradiography.

LigDs are proposed to act on DNA gapped substrates that can arise during the limited base pairing realignment of the DNA ends produced by DSBs [18], [25]. To analyze the *Bsu*LigD filling-in ability, DNA molecules harboring gaps of 1–5 nucleotides were used as substrates of the polymerization activity. In addition, the influence of a phosphate group at the 5′ end of the downstream strand in the *Bsu*LigD gap-filling efficiency was also evaluated. As shown in Figure 2 (left panels), in the presence of a downstream 5′-OH end first ribonucleotide addition was higher than that of dNMPs, regardless of the length of the gap. Notwithstanding *Bsu*LigD accomplished efficient filling of the 2-nt gap with NTPs, it mainly inserted only one NMP when acting on longer gaps. These results would be pointing to gaps of 1–2 nt as the preferred substrates of the enzyme, suggesting a simultaneous binding of LigD to the upstream and downstream regions of the DNA substrate. Therefore, the longer the gap, the lower the insertion efficiency, maybe due to the loss of downstream contacts. In this sense, the binding of LigD to the downstream strand in the 2-nt gap molecule could be aiding the second NMP addition observed to occur with this molecule. The presence of a 5′-phosphate at the distal margin of the gap stimulated insertion of dNMPs principally in the 2-nt gap molecules (Figure 2, right panel) pointing to a specific recognition of this group by the enzyme. This result would represent an extension to the rest of LigDs of previous data obtained with *Mycobacterium tuberculosis* LigD (*Mtu*LigD) whose templated polymerization reaction was greatly stimulated by a 5′-P group [31]. The crystal structure of the polymerase domain of *Mtu*LigD mediating the synapsis of two non-complementary DNA ends showed the enzyme making intimate contact with the upstream primer portion and with the 5′-P on the downstream strand through two Lys residues absolutely conserved in NHEJ LigDs [26], [42] and whose homologs in *Bsu*LigD would correspond to Lys331 and Lys341 (see Figure 1C).

### *Bsu*Ku interacts functionally with *Bsu*LigD enhancing its gap-filling efficiency

Both, eukaryotic and prokaryotic Ku have been described to bind dsDNA with high affinity [8], [19], [44]. In the current working model of bacterial NHEJ, Ku recognizes and binds to the broken ends, recruiting LigD that further catalyzes the sealing step of break repair [18], [25], [26].

**Figure 3:**
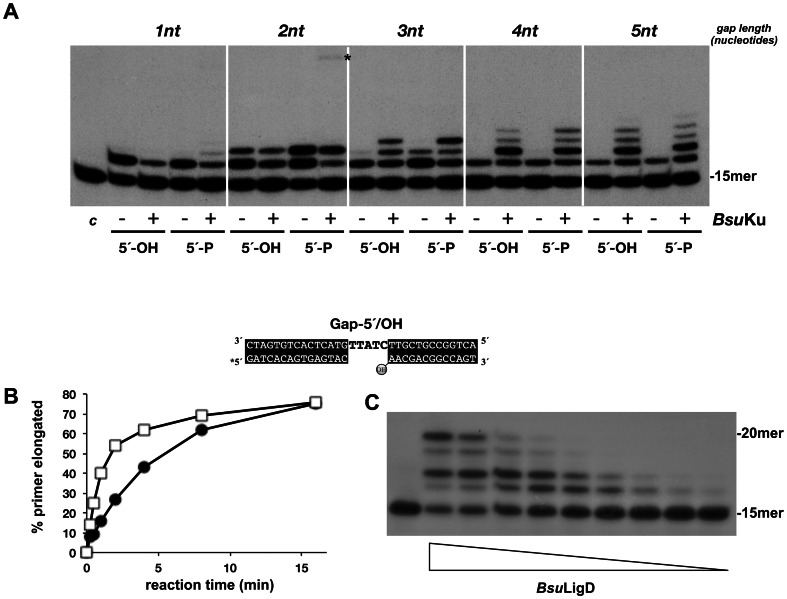
*Bsu*Ku interacts functionally with *Bsu*LigD enhancing its gap-filling efficiency. (A) Effect of *Bsu*Ku on the gap-filling efficiency of *Bsu*LigD. The assay was performed as described in Materials and Methods, incubating 0.4 ng of *Bsu*LigD with 2.5 nM of the indicated gapped molecule, 5 mM MnCl_2_ and 1 µM NTPs, in the absence (−) or presence (+) of 40 ng of *Bsu*Ku. The 5′ end of the downstream strand is specified (5′-OH, 5′-P). After incubation for 10 min at 30°C, samples were analyzed by 8 M urea and 20% PAGE and autoradiography. (B) *Bsu*Ku increases *Bsu*LigD primer usage. The assay was performed as described in Materials and Methods, incubating 10 ng of *Bsu*LigD with 1 nM of the depicted gapped molecule, 5 mM MnCl_2_ and 10 µM NTPs, in the absence (full circles) or presence (squares) of 40 ng of *Bsu*Ku. After incubation for the indicated times at 30 °C, samples were analyzed by 8 M urea and 20% PAGE and autoradiography and the unextended and elongated primer molecules quantified using a Molecular Dynamics PhosphorImager. Percentage of extended primers (elongated/elongated+unextended) was plotted against reaction time. (C) *Bsu*Ku does not prevent *Bsu*LigD dissociation after each NMP insertion reaction. The assay was carried out as described in Materials and Methods, using the 5-nt gapped molecule depicted, in the presence of 40 ng of *Bsu*Ku, 10 µM NTPs and decreasing amounts of *Bsu*LigD (2.5, 1.25, 0.6, 0.3, 0.15, 0.07, 0.04, 0.02 and 0.01 ng). After incubation for 20 min at 30°C, samples were analyzed by 8 M urea-20% PAGE and autoradiography.

To study whether *Bsu*Ku exerts a functional influence on *Bsu*LigD activity, the gap-filling efficiency of the latter was examined in the presence or absence of *Bsu*Ku (see Figure 3A). As it can be observed, *Bsu*Ku slightly impaired the primer usage by *Bsu*LigD when acting on the 1-nt gapped molecule. This result could indicate that both proteins cannot accommodate simultaneously in such a short gap. Conversely, *Bsu*Ku enhanced *Bsu*LigD polymerization efficiency on gaps longer than one nucleotide, irrespective of the identity of the 5′-group of the downstream DNA, increasing the proportion of products longer than 1 nucleotide on the 2- and 3-nt gaps. Interestingly, in the presence of *Bsu*Ku the NMP insertion catalyzed by *Bsu*LigD was not halted after the first nucleotide addition and the enzyme performed gap filling to completion. Similar results were observed with the 4- and 5-nt gap molecules on which *Bsu*Ku enhanced gap filling efficiency of *Bsu*LigD. Interestingly, although *Bsu*Ku caused a nearly 3-fold improvement of the primer usage by *Bsu*LigD (Figure 3B), it did not prevent dissociation of the enzyme after each NMP insertion, as the length of the polymerization products decreased with the enzyme/DNA ratio (see Figure 3C). These results led to propose that *Bsu*Ku promotes a fast recruitment of *Bsu*LigD to the 3′ ends increasing its polymerization efficiency, and suggest that, as proposed for *P. aeruginosa*, utilization of ribonucleotides for DNA synthesis during NHEJ in *B. subtilis* could be restricted to short repair tracts [38], [41].

**Figure 4:**
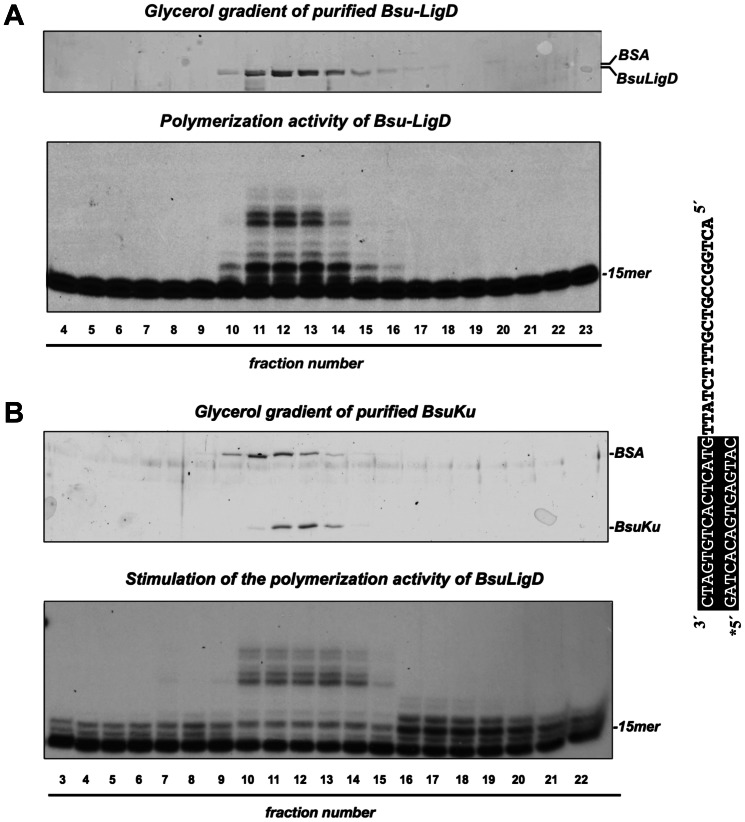
Cosedimentation of polymerization activity with *Bsu*LigD. (A) Top panel shows a SDS-PAGE analysis followed by Coomassie Blue staining of gradient fractions 4–23 collected after sedimentation of the purified *Bsu*LigD on a 15–30% glycerol gradient (see Materials and Methods). Bottom panel shows the polymerization products obtained after incubating for 20 min at 30°C 4 µl of each fraction with the template/primer structure depicted on the right (see Materials and Methods), in the presence of 5 mM MnCl_2_, 100 µM dNTPs and 50 ng of *Bsu*Ku. Asterisk indicates the ^32^P 5′-labeled end of the primer strand. (B) Stimulation of the *Bsu*LigD polymerization activity cosediments with *Bsu*Ku. Top panel shows a SDS-PAGE analysis followed by Coomassie Blue staining of gradient fractions 3–22 collected after sedimentation of the purified *Bsu*Ku on a 15–30% glycerol gradient (see Materials and Methods). Bottom panel shows the polymerization products obtained after incubating for 20 min at 30°C 50 ng of *Bsu*LigD with the template/primer structure depicted on (A) (see Materials and Methods), in the presence of 5 mM MnCl_2_, 100 µM dNTPs and 4 µl of each fraction.

As a control to ensure proper assignment of the polymerization activity to *Bsu*LigD and its stimulation by *Bsu*Ku, the purified proteins were individually sedimented through a glycerol gradient, and the collected fractions assayed accordingly (see Materials and Methods). Thus, *Bsu*LigD fractions were evaluated for DNA polymerase activity on a primer/template substrate in the presence of *Bsu*Ku (see Materials and Methods). As shown in Figure 4A, the single activity peak observed coincided with the protein peak, identified by Coomassie Blue staining after SDS-PAGE analysis of each gradient fraction. These results allowed to ascribe the DNA polymerization activity to *Bsu*LigD, ruling out the presence of a contaminant DNA polymerase from *E. coli*. Similarly, each *Bsu*Ku fraction was analyzed in its ability to stimulate *Bsu*LigD polymerization activity on the same substrate (see Figure 4B). As it is shown, stimulation of the polymerization reaction was observed with those fractions containing the maximal amount of protein, a result that led us to conclude that the improvement of *Bsu*LigD activity is attributable to *Bsu*Ku.

Altogether these experimental evidences suggest i) that the complex *Bsu*Ku/*Bsu*LigD is well suited and specialized to fill short gaps 2–3 nucleotides long, mainly those bearing a P group at the 5′ end of the downstream strand, and ii) the existence of a functional interaction between *Bsu*Ku and *Bsu*LigD.

### Ligase activity of *Bsu*LigD enables the enzyme to catalyze complete repair of a gapped DNA substrate

**Figure 5:**
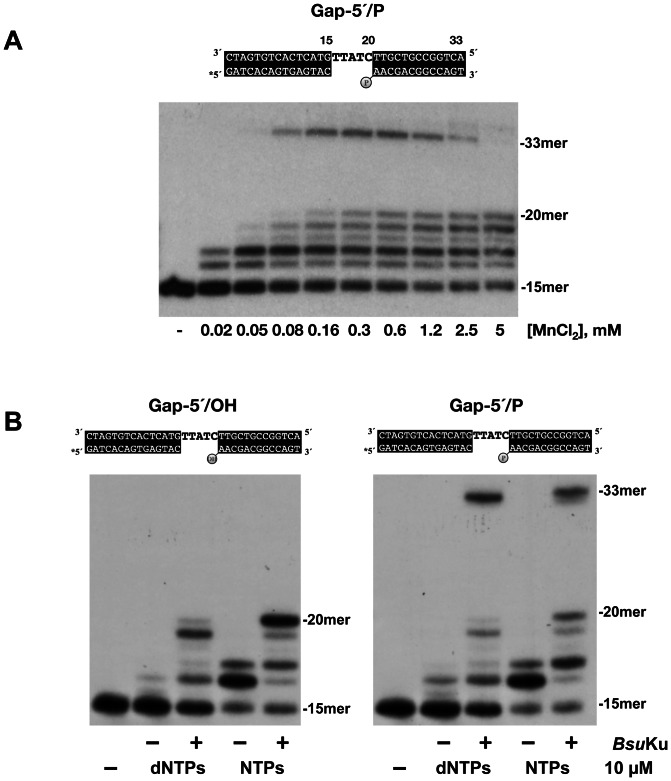
Complete repair of a gapped DNA substrate by the *Bsu*LigD/*Bsu*Ku complex. (A) Optimal metal requirement to couple polymerization and ligation activities of *Bsu*LigD/*Bsu*Ku complex. The gap-filling assay was performed as described in Materials and Methods by incubating 50 ng of *Bsu*LigD and *Bsu*Ku with 1.5 nM of the gapped DNA molecule depicted on top, in the presence of the indicated concentrations of MnCl_2_ and 1 µM NTPs. After incubation for 10 min at 30 °C, the reactions were stopped by adding EDTA up to 10 mM. Samples were analyzed by 8 M urea, 20% PAGE and autoradiography. Asterisk indicates the 5′^32^P-labeled end of the primer strand. (B) Polymerization and ligase activities of *Bsu*LigD allow complete repair of a gapped molecule. The different molecules used in the analysis are depicted on top of each panel. The assay was performed as described in Materials and Methods by incubating the indicated gapped substrates depicted on top of the figure with 10 ng of *Bsu*LigD, 10 µM of either dNTPs or NTPs, 0.6 mM MnCl_2_, in absence (−) or presence (+) of 40 ng of *Bsu*Ku. After incubation for 20 min at 30°C, the primer-extended products corresponding either to the filling-in reaction or to the complete repair reaction (filling-in + ligation) were analyzed by 8 M urea-20% PAGE and autoradiography.

As it can be observed in Figure 3A, with the 2 nt gap-5′/P substrate and in the presence of *Bsu*Ku, *Bsu*LigD yielded a faint band (marked with an asterisk) that would correspond to a small proportion of the +2 product ligated to the downstream strand, supporting the prediction of a ligase activity in this enzyme [24]. Accordingly, *Bsu*LigD catalyzed the metal dependent formation of a radiolabeled covalent ligase-adenylate adduct in the presence of [α-^32^P]ATP, the initial step in DNA ligation, which comigrated with the *Bsu*LigD in a SDS-PAGE (see Figure S2). The coincidence of the maximal ligase activity, assayed on a nicked DNA molecule, with the mass peak fractions obtained from the glycerol gradient sedimentation of the purified protein shown in Figure 4A, corroborated the proper assignment of the ligase activity to *Bsu*LigD (Figure S3). The low proportion of the ratio between the ligated and the +2 products aforementioned led to analyze the optimal metal requirement to couple the polymerization and ligation activities. Therefore, *Bsu*LigD/*Bsu*Ku complex was incubated with the 5-nt gapped molecule in the presence of increasing concentrations of Mn^2+^ as metal activator and a higher amount of *Bsu*LigD to augment the quantity of elongated +5 products. As it can be observed in Figure 5A, *Bsu*LigD filled the gap and sealed the resulting nick to the 5′-P terminus of the downstream strand, giving rise to the 33-mer repaired product, the MnCl_2_ concentration required to get the maximal ligation/+5 product ratio ranging between 0.16 and 1.2 mM, in good correlation with the *in vivo* levels of Mn^2+^ within the cell (0.3 mM; [45]). At 5 mM MnCl_2_ the amount of ligation products was negligible, a result that would account for the low proportion of repaired products observed with the 2-nt gapped molecule previously mentioned. As shown in Figure 5B the presence of a downstream 5′-P group and *Bsu*Ku was absolutely required to allow *Bsu*LigD to repair totally the 5-nt gap, with either dNTPs or NTPs.

### Mismatched 3′ termini elongation on gapped molecules

**Figure 6:**
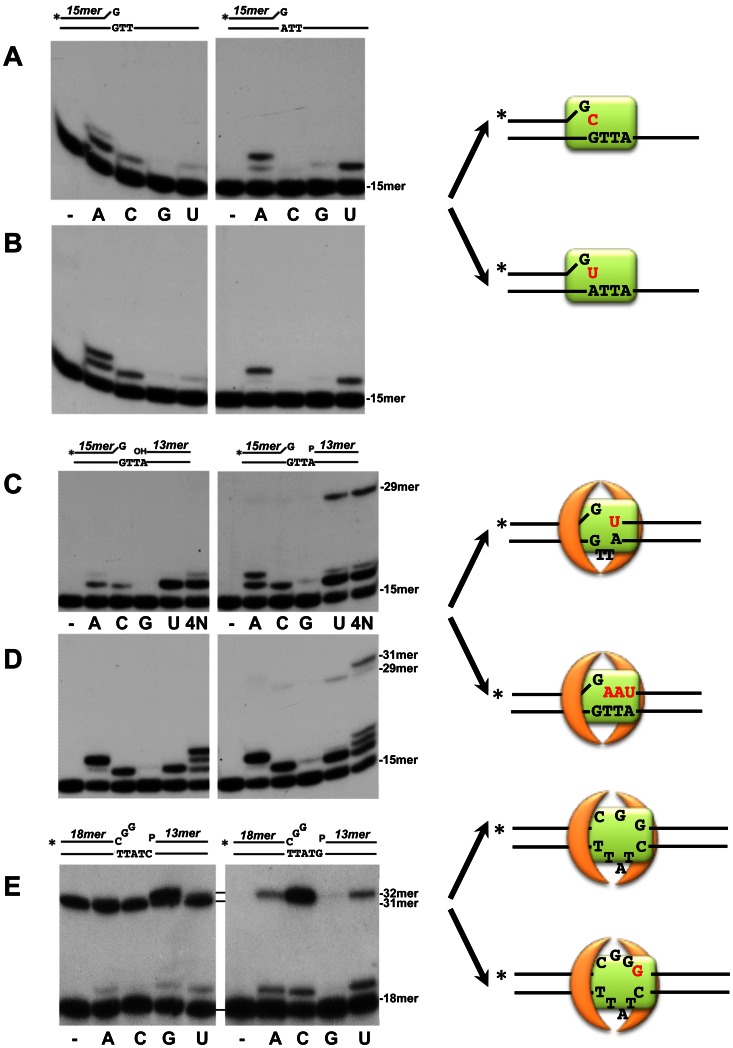
Activity of *Bsu*LigD/Ku complex on molecules bearing a mispaired 3′ end. (A) and (B) Elongation of dG:dG and dG:dA mispairs present in a template/primer structure. The assay was performed essentially as described in Materials and Methods in the presence of 1 µM of the indicated nucleotide, 0.6 mM MnCl_2_, 1.5 nM of the depicted substrate and 50 ng of *Bsu*LigD, in the absence (A) or presence (B) of 50 ng of *Bsu*Ku. After incubation for 20 min at 30°C the elongation and ligation products were analyzed by 8 M urea-20% PAGE and autoradiography. Position of the unextended primer is indicated on the right. Asterisks indicate the ^32^P 5′-labeled end of the primer strand. (C) and (D) Activity on a mispaired 3′ end in the 3 nucleotides gapped molecule. The assay was performed essentially as described in Materials and Methods in the presence of 1 µM of the indicated nucleotide (4N: the four nucleotides), 0.6 mM MnCl_2_, 1.5 nM of the depicted gapped substrate and 50 ng of *Bsu*LigD, in the absence (C) or presence (D) of 50 ng of *Bsu*Ku. After incubation for 20 min at 30°C the elongation and ligation products were analyzed by 8 M urea-20% PAGE and autoradiography. Position of the unextended primer and ligation products is indicated on the right. (E) Activity on a 3 nucleotides mismatched 3′ terminus present in 5 nucleotides gapped molecules. The assay was performed essentially as described in Materials and Methods in the presence of 1 µM of the indicated nucleotide, 0.6 mM MnCl_2_, 1.5 nM of the depicted gapped substrate, 50 ng of *Bsu*LigD and 50 ng of *Bsu*Ku. After incubation for 20 min at 30 °C the elongation and ligation products were analyzed by 8 M urea-20% PAGE and autoradiography. Position of the unextended primer and ligation products is indicated on the right. The two DNA substrate molecules only differ in the marginal nucleotide of the template region. Schematic representations of the proposed realignments of the primer and template strands are drawn on the right. *Bsu*Ku homodimer and *Bsu*LigD are represented by orange arches and a green rectangle, respectively.

*Bsu*LigD/Ku complex should deal *in vivo* with potential mispairings resulting during the rejoining of non-compatible DNA ends. Therefore, it was of interest to test the capability of *Bsu*LigD/Ku complex to elongate mismatched 3′ termini. As it can be observed in Figure 6A (left panel), in the presence of the next correct ATP, *Bsu*LigD could elongate the dG:dG mispair by adding two AMP residues opposite the two consecutive dTMPs of the template. Interestingly, the enzyme could also incorporate the "incorrect" CMP residue to the mispaired 3′ end, a result that could be indicative of an intrinsic ability of *Bsu*LigD to slip the primer-terminus back to catalyze CMP insertion opposite the dGMP of the mispair. Accordingly, *Bsu*LigD could extend the dG:dA mispair by adding either AMP or UMP at the same extent (Figure 6A, right panel). As it is observed in Figure 6B, the presence of *Bsu*Ku did not prevent the primer slippage. Interestingly, previous studies of *Pae*LigD showed that the polymerization domain could insert two consecutive TMPs opposite a template dA-dT dinucleotide [40]. This result led to conclude that *Pae*LigD accomplished misincorporation of TMP directed by the templating dT. However, based on the results presented here, an alternative and non-exclusive possibility would be that the templating dA directed the iterative addition of two TMP residues, a fact that would substantiate the ability to slip the primer terminus back as a general signature of bacterial LigDs.

Elongation of the mispaired 3′ terminus was also evaluated in a 3-nt gapped molecule, as these substrates would mimic NHEJ intermediates, namely the bridging step of an end joining reaction, as described [18]. As it can be observed in Figure 6C *Bsu*LigD recognized specifically the downstream 5′-P group also when acting on these substrates. In addition, irrespectively of the 5′ group, the presence of a downstream strand did not prevent the primer slippage described above, as inferred from the CMP insertion. Additionally, on this substrate *Bsu*LigD also inserted a UMP residue very proficiently, a proportion of the UMP-extended primers being further sealed to the downstream 5′P group to give rise to a 29 mer ligation product. This result suggests that, as it occurs with other bacterial LigDs [31], [40], *Bsu*LigD can use as template of its polymerization reaction the marginal dA by flipping out of the catalytic site the two intermediate dTs of the template. The resulting matched base pair is further ligated to the downstream 5′P end causing a two nucleotides deletion mutation (see scheme on the right). It was significant that on this substrate the presence of the four NTPs did not prevent the preferential insertion and further ligation of UMP opposite the marginal dA of the template. However, *Bsu*Ku compelled *Bsu*LigD to fill the gap to completion following the 3′-5′ template instructions, guaranteeing a minimal lost of the original sequence and giving rise to the final 31 mer ligation product (see Figure 6D).

Similar experiments were also carried out in the presence of a 3 nucleotides mismatched 3′ terminus (see Figure 6E). As it is shown, when acting on the 5-nt gapped molecule, and in the absence of nucleotides, *Bsu*LigD gave rise to a 31 mer band. The formation of this product would indicate a direct pairing of the 3′ G to the marginal dC of the template and further ligation to the downstream strand, yielding a two nucleotides deletion (see left panel Figure 6E). Additionally, when the nucleotides were supplied independently, the enzyme inserted specifically G opposite the marginal dC, producing a final 32 mer ligated product. As expected, substitution of the marginal dC of the template by a dG precluded *Bsu*LigD to promote direct ligation of the 3′ and 5′ ends (see right panel and scheme in Figure 6E), and now C was the nucleotide mainly inserted. Thus, it seems that to get final ligation, *Bsu*LigD polymerization active site would be designed to accommodate the "bubble" formed by a double or a triple mispairing to juxtapose the upstream 3′ and the downstream 5′ termini. Under these conditions a low proportion of misinserted and further ligated A and U was also observed suggesting that, despite its potential mutagenicity, the enzyme would cope with mispaired nucleotides at the joining site to restore the integrity of the broken DNA strand.

## Conclusions

Here, it has been demonstrated that *B. subtilis* gene *ykoU* encodes for a LigD that shares with other well documented members of this family the essential biochemical features to repair DNA breaks [18], [25], [31], [40]. Thus, *Bsu*LigD i) has a polymerization activity, ii) shows a preferential insertion of rNMPs, iii) recognizes specifically a phosphate group at the downstream 5′ end of gapped DNA molecules, iv) contains an intrinsic ligase activity and v) it can promote dislocations of the template and primer strands, suggesting the high flexibility of the catalytic site to deal with and hold different and unstable DNA realignments. These biochemical characteristics would have evolved to allow the enzyme to play the crucial joining of the two broken strands, despite of its potential mutagenicity.

*Mtu*- and *Pae*-LigDs contain, besides a polymerization and ligase domain, a 3′-phosphoesterase (PE) domain with a nucleolytic activity that would remodel the 3′ modified ends prior to their elongation and further ligation [18], [25]. Thus, PE domain of *Pae*LigD has been shown to hydrolyze 3′-phosphates from the DSB to produce a 3′-OH primer for the polymerization activity. In addition, the enzyme can also degrade the RNA tract, synthesized by the polymerization activity, to yield a 3′-NMP, the preferred substrate of its ligase activity [41], [46], [47]. The PE domain of *Mtu*LigD can also resect nucleotide flaps often produced by microhomology-mediated synapsis [20]. The potentiality of bacterial Ku protein to recruit proteins other than LigD has been recently demonstrated with the identification of the DNA repair helicase UvrD1 and Sir2-like protein as novel interaction partners for mycobacterial Ku [27], [28]. This finding, together with the absence of a PE domain in *Bsu*LigD opens the possibility of DNA end-cleaning protein recruitment by *Bsu*Ku to the DSB. In this sense, several *B. subtilis* proteins have been proposed to play a role in basal end processing [48] as *B. subtilis* SdcD whose ortholog in *Escherichia coli* has a nucleolytic activity [49] and forms a complex with the *B.subtilis* SbcC protein shown to participate in the repair of DSB [50]. Moreover, Mre11 protein from the MRX complex required for NHEJ in yeast is a nuclease of the SbcCD family shown to interact with Yku80 [1]. Another protein that could take part in processing 3′ ends during NHEJ is *B. subtilis* polynucleotide phosphorylase (PNPase) as it achieves Mn^2+^ dependent 3′-5′ phosphorolytic degradation on 3′-tailed duplex DNAs [51], a null *B. subtilis pnpA* mutation being epistatic to the Δ*ku* mutation. Finally, an attractive candidate would be *B. subtilis* polymerase X (PolX) as this DNA repair protein is endowed with a 3′-5′ exonuclease activity specialized in resecting 3′ flap structures [52] and it can sanitize 3′ damaged ends [53]. The potential involvement of bacterial PolX in NHEJ would agree with the increased sensitivity against DSBs provoked by γ-rays observed in the bacterium *D. radiodurans* upon deletion of the gene that codes for its own PolX [54], and with the certainty that in eukaryotes the gap filling step during NHEJ is carried out by a family X DNA polymerase [2], [3], [12]. Thus, the probable recruitment and involvement of a trans-acting nucleolytic activity to remodel 3′ ends before the repair of DSBs would imply that bacterial NHEJ machinery could be more complex than currently thought.

## Supporting Information

Figure S1 **Organization of the different enzymatic activities of** ***Bsu*** **LigD and representatives of the two LigD subfamilies described in** [**26**] **.** The polymerization (Pol), phosphoesterase (PE) and ligase domains are represented as green, yellow and orange cilinders, respectively.
(TIF)

Figure S2 ***Bsu*** **LigD-adenylate complex formation.** Reactions were performed as described in Materials and Methods by incubating the indicated amount of purified *Bsu*LigD with 5 µM [α-^32^P]ATP (2.5 µCi) in the presence of 5 mM MnCl_2_. After incubation for 10 min at 30°C the reaction was stopped by adding 10 mM EDTA and 0.1% SDS. The samples were then filtered through Sephadex G-50 spin columns to remove the non-incoporated ATP, and further analyzed by 12% SDS-PAGE. Label transfer to the 70-kDa *Bsu*LigD polypeptide was visualized by autoradiography of the dried gel. The position of the BsuLigD-AMP complex is indicated on the left.
(TIF)

Figure S3 ***Bsu*** **LigD has an inherent ligase activity.** The assay was performed by incubating 4 µl of each fraction collected after sedimentation of the purified *Bsu*LigD on a 15–30% glycerol gradient (see Materials and Methods) with 1.5 nM of the nicked DNA depicted (asterisk indicates the 5′^32^P-labeled end of the primer strand) in the presence of 20 µM MnCl_2_ and 50 ng of *Bsu*Ku. After incubation for 10 min at 30°C the ligation products were analyzed by 8 M urea-20% PAGE and autoradiography.
(TIF)
