# Supplementary figures and images for: The Minimal Bacillus subtilis Nonhomologous End Joining Repair Machinery
Source: PLoS One. 2013 May 17;8(5):e64232. doi: 10.1371/journal.pone.0064232 (PMC3656841; doi:10.1371/journal.pone.0064232)

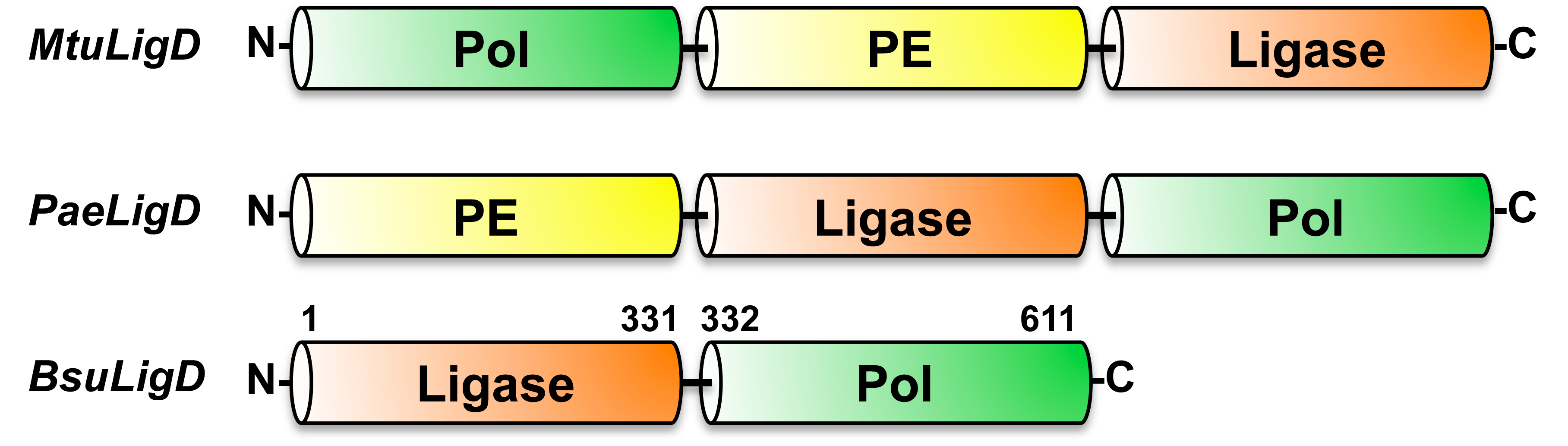

Supplement: Figure S1 — Organization of the different enzymatic activities of Bsu LigD and representatives of the two LigD subfamilies described in [26] . The polymerization (Pol), phosphoesterase (PE) and ligase domains are represented as green, yellow and orange cilinders, respectively. (TIF) [file pone.0064232.s001.tif]

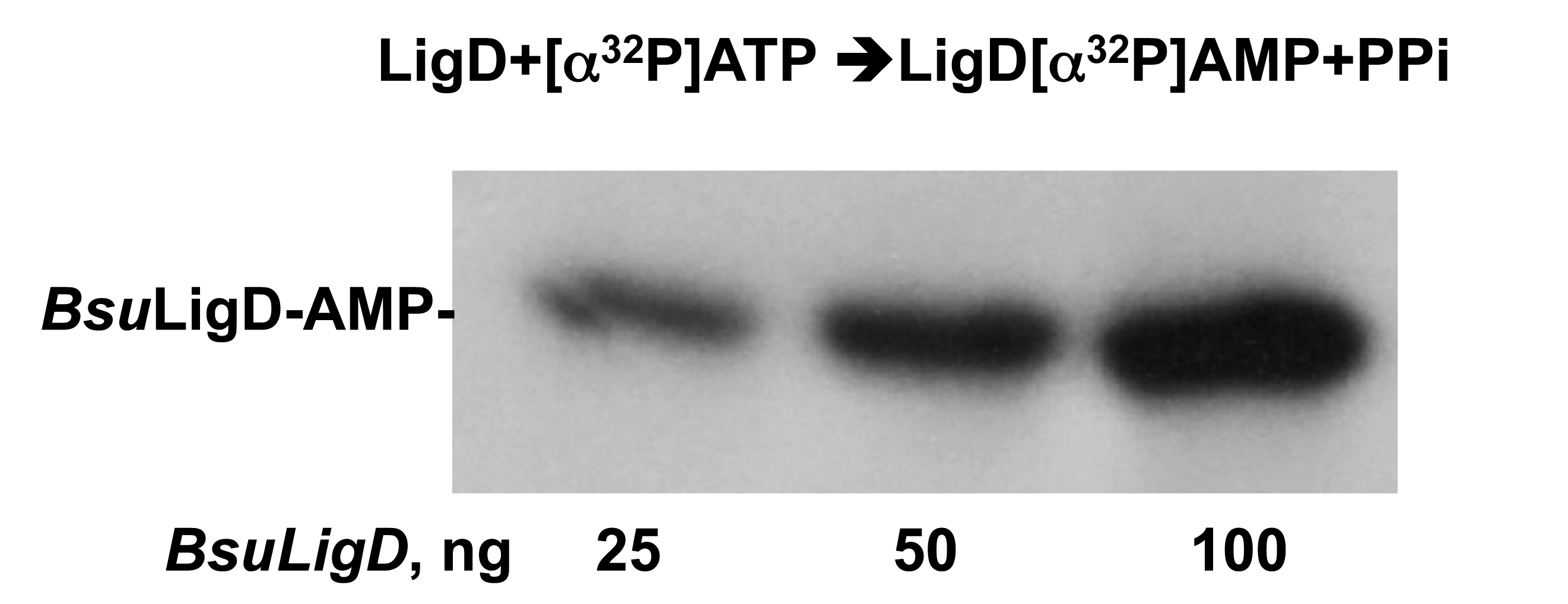

Supplement: Figure S2 — Bsu LigD-adenylate complex formation. Reactions were performed as described in Materials and Methods by incubating the indicated amount of purified BsuLigD with 5 µM [α-32P]ATP (2.5 µCi) in the presence of 5 mM MnCl2. After incubation for 10 min at 30°C the reaction was stopped by adding 10 mM EDTA and 0.1% SDS. The samples were then filtered through Sephadex G-50 spin columns to remove the non-incoporated ATP, and further analyzed by 12% SDS-PAGE. Label transfer to the 70-kDa BsuLigD polypeptide was visualized by autoradiography of the dried gel. The position of the BsuLigD-AMP complex is indicated on the left. (TIF) [file pone.0064232.s002.tif]

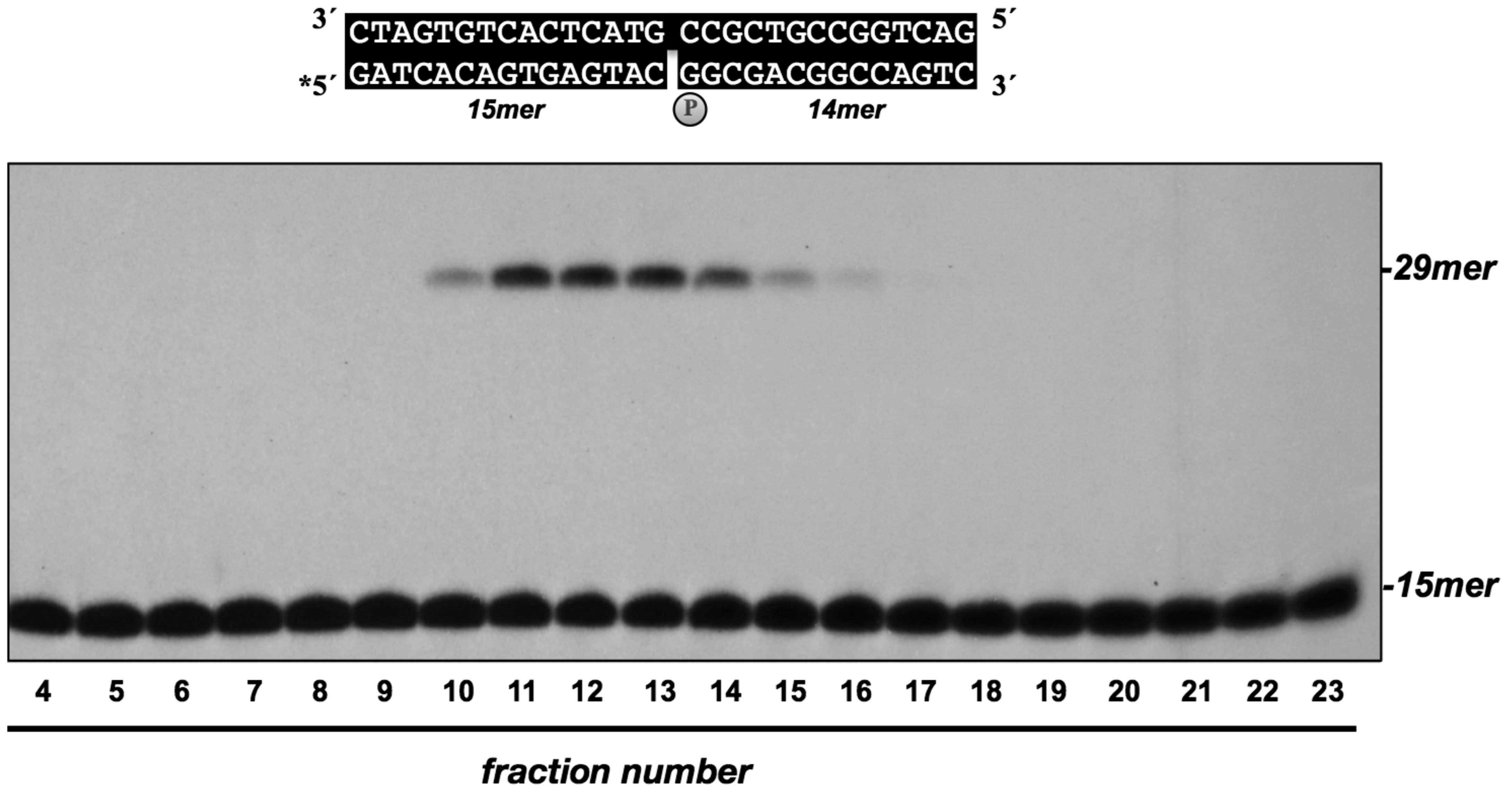

Supplement: Figure S3 — Bsu LigD has an inherent ligase activity. The assay was performed by incubating 4 µl of each fraction collected after sedimentation of the purified BsuLigD on a 15–30% glycerol gradient (see Materials and Methods) with 1.5 nM of the nicked DNA depicted (asterisk indicates the 5′32P-labeled end of the primer strand) in the presence of 20 µM MnCl2 and 50 ng of BsuKu. After incubation for 10 min at 30°C the ligation products were analyzed by 8 M urea-20% PAGE and autoradiography. (TIF) [file pone.0064232.s003.tif]
